# History-dependent percolation on multiplex networks

**DOI:** 10.1093/nsr/nwaa029

**Published:** 2020-02-20

**Authors:** Ming Li, Linyuan Lü, Youjin Deng, Mao-Bin Hu, Hao Wang, Matúš Medo, H Eugene Stanley

**Affiliations:** Department of Thermal Science and Energy Engineering, University of Science and Technology of China, Hefei 230026, China; Institute of Fundamental and Frontier Sciences, University of Electronic Science and Technology of China, Chengdu 610054, China; Alibaba Research Center for Complexity Sciences, Hangzhou Normal University, Hangzhou 310036, China; Beijing Computational Science Research Center, Beijing 100193, China; Hefei National Laboratory for Physical Sciences at Microscale, Department of Modern Physics, and CAS Center for Excellence and Synergetic Innovation Center in Quantum Information and Quantum Physics, University of Science and Technology of China, Hefei 230026, China; Department of Thermal Science and Energy Engineering, University of Science and Technology of China, Hefei 230026, China; Institute of Fundamental and Frontier Sciences, University of Electronic Science and Technology of China, Chengdu 610054, China; Institute of Fundamental and Frontier Sciences, University of Electronic Science and Technology of China, Chengdu 610054, China; Alibaba Research Center for Complexity Sciences, Hangzhou Normal University, Hangzhou 310036, China; Department of Physics and Center for Polymer Studies, Boston University, Boston, MA 02215, USA

**Keywords:** percolation, multiplex networks, critical phenomena, brain networks

## Abstract

The structure of interconnected systems and its impact on the system dynamics is a much-studied cross-disciplinary topic. Although various critical phenomena have been found in different models, study of the connections between different percolation transitions is still lacking. Here we propose a unified framework to study the origins of the discontinuous transitions of the percolation process on interacting networks. The model evolves in generations with the result of the present percolation depending on the previous state, and thus is history-dependent. Both theoretical analysis and Monte Carlo simulations reveal that the nature of the transition remains the same at finite generations but exhibits an abrupt change for the infinite generation. We use brain functional correlation and morphological similarity data to show that our model also provides a general method to explore the network structure and can contribute to many practical applications, such as detecting the abnormal structures of human brain networks.

## Teaser

We propose a unified framework for studying the origin of the discontinuous transitions of the percolation process on interacting networks,whichalsohas wide practical applications in network science.

## INTRODUCTION

Our understanding of percolation properties of networks has expanded significantly in recent years. Percolation theory, a classical model in statistical physics, has been applied in a number of different network science topics, such as network structure [1,2], network robustness [3–5], node ranking [6] and community detection [7], as well as in studies of network dynamics, such as information spreading [8], and highway traffic flows [9].

Although we often assume that the underlying structure of a network is complex, the rules for the percolation process are comparatively simple. Typically, each link or node is occupied with a given probability *p*, independent of the states of other links and nodes. By contrast, real-world network processes are often ‘history-dependent’: for example, the spread of a particular disease can depend on the spread of other diseases [10] and it can also be influenced by the availability of immunization information [11]. Network topology itself can be affected by cascading failures [12,13] or by recovery processes [14] in other networks. The universality class of a percolation transition depends on the quenched disorder topology induced by the previous percolation transition [15].

A second complication results from the presence of multiple interaction channels that are often involved in history-dependent processes [16,17]. Such systems can be naturally described in terms of multiplex networks where nodes are connected through different types of links [18], such as the social networks with different types of interactions which can be either online or offline [19,20], the multilayer transportation network with various means of vehicles [21], and the brain network with both functional correlation and morphological similarity [22]. One of the typical examples of iterative interactions on multiplex networks is the interplay between the spreading of an epidemic and the information awareness that prevents its further spreading [20]. Another example is that of cascading failures on coupled networks of power distribution and communications [12,23]. Although these works consider a similar mechanism (i.e. a history-dependent process), the former features a continuous percolation transition, while the latter features a discontinuous phase transition [24]. We address the question of whether there is a general model of history-dependent percolation on multiplex networks where both continuous and discontinuous percolation transitions can emerge.

To answer this question, we introduce an iterative percolation model on multiplex networks. The percolation of each generation is based on the resulting state of the previous generation, which is referred to as history-dependent percolation here. Different to previous percolation models with some iterative processes, our work focuses also on the intermediate states of the iterative process; these intermediate states are referred to as generations here. The benefits of doing so are twofold. First, while individual intermediate generations can have their direct real counterparts, they are overlooked by focusing on the infinite (steady-state) generation. Second, by examining generations in succession, we gain understanding of the origin of the discontinuous transition in the steady state and its relation with the continuous transition.

Theoretical analysis indicates that the intermediate states of the recursive process are not cluttered, hence the percolation transition can be observed in any generations. Monte Carlo simulations on Erdős–Rényi (ER) networks further suggest that all these continuous transitions belong to the same universality class. Although the size of the giant cluster becomes smaller and smaller as the generations progress, endless iterations cannot completely destroy the network when it is initially dense enough. Instead, a non-vanished cluster suddenly appears above the threshold indicating a discontinuous percolation transition. Specifically, scale-free (SF) networks with exponent 2 < γ < 3 have a vanished critical point for any finite generations, and the non-trivial critical point can suddenly emerge when the number of percolation generations diverges. With the example of human brain networks, our model shows that to find a meaningful structure (such as the abnormal structures of human brain networks), it is not always necessary to evolve the recursive process into the steady states. Our model thus provides a novel approach to analyse the network structure.

## RESULTS

### History-dependent model

**Figure 1.**
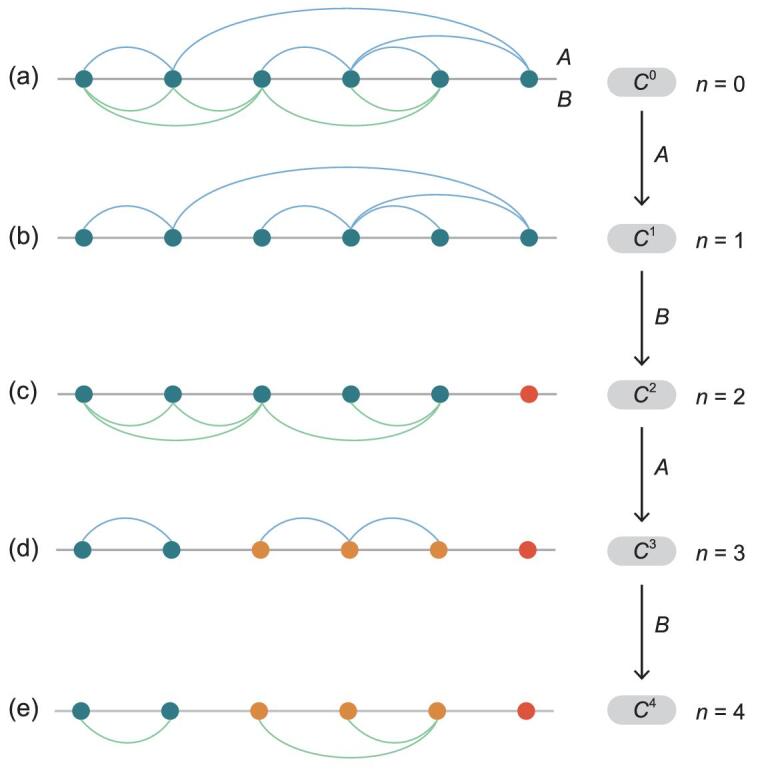
Sketch of history-dependent percolation on a small multiplex network. (a) A multiplex network with two layers *A* and *B* separated by the horizontal gray line. (b) Generation *n* = 1. The configuration *C*^1^ induced by layer *A* is connected. (c) Generation *n* = 2. The configuration induced by layer *B* on *C*^1^ has two clusters indicated by blue and red. (d) Generation *n* = 3. The configuration *C*^3^ induced by layer *A* on *C*^2^ has three clusters indicated by blue, orange and red, respectively. (e) Generation *n* = 4. Applying layer *B* on configuration *C*^3^, no new clusters can be found; the percolation process has reached a steady state. If the system is large enough, this process can be done to any number of generations.

An undirected multiplex network is formed by a set of *N* nodes and multiple layers with links. Each layer is described by its adjacency matrix, whose unit elements correspond to links between the corresponding nodes. For simplicity and without loss of generality, we consider here only the case of a multiplex network with two layers, which we refer to as layers *A* and *B*, respectively (see Fig. 1a as an example). An extension to the general case with more layers is straightforward, and some discussion on this can be found in the Supplementary Information.

To model the history-dependent iterative process, we use the two network layers alternately to investigate the percolation process in every generation. For generation *n* = 1, we use layer *A* to check the percolation process among all the nodes. Then, nodes form a configuration *C*^1^, see Fig. 1b. The percolation of generation *n* = 2 is induced by layer *B* based on the configuration *C*^1^ formed in generation *n* = 1. Specifically, if two nodes *i* and *j* who are in the same cluster in *C*^1^ are connected in layer *B*, then they will be connected in this generation. Traversing all node pairs in the same clusters, we obtain a new configuration *C*^2^, see Fig. 1c. Similarly, for generation *n* = 3, we use layer *A* to perform a percolation on configuration *C*^2^. If two nodes *i* and *j* who are in the same cluster in *C*^2^ are connected in layer *A*, they will be connected in this generation. Then, a new configuration *C*^3^ is obtained, see Fig. 1d, and so on, to any number of generations. Note that the two layers *A* and *B* are used cyclically in this progressive process.

Note that once the initial configuration of the two layers is given, the constructions of clusters in all generations are deterministic. To study the percolation transition in each generation, a natural choice of the control parameter is the average degree $z$ of the initial network layers. For a network ensemble with a fixed average degree (or for a given real multiplex network), one can study the percolation transition by introducing the link occupation probability *p* as the control parameter: the fraction 1 − *p* of links in each layer are chosen at random and removed, and the remaining links are then used in the iterated percolation processes.

### Network with ER layers

**Figure 2.**
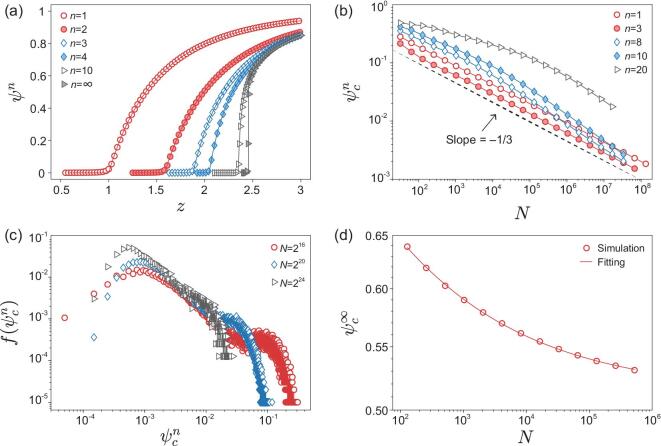
The simulation results for ER networks. (a) The size of the giant cluster ψ^*n*^ as a function of the average degree $z$ for different generations. The solid lines are the theoretical results obtained by our method. The system size in simulations is *N* = 2^16^. (b) The order parameter at the critical point $\psi _c^n$ for finite generations as a function of the network size *N*. The simulation results are obtained by averaging over all the model realizations. (c) The distribution of the order parameters obtained in each individual realization $f(\psi _c^n)$ for generation *n* = 10. (d) The order parameter at the critical point $\psi _c^\infty$ for the infinite generation as a function of the network size *N*. The simulation results are the average over the roughly $60\%$ of model realizations that percolate. The fitted curve has the form $\psi _c^{\infty }= \psi _{c0}^\infty +O(N^{-\varepsilon })$ with $\psi _{c0}^\infty =0.514\pm 0.001$ and ϵ = 0.233 ± 0.005.

We first consider the case where the two network layers are both ER networks with the same average degree $z$, for which the model can be solved exactly (see Methods). The results indicate that the first generation and also all finite generations of iterative percolation demonstrate a continuous percolation transition. Figure 2a shows that the computed order parameter ψ^*n*^ agrees well with the numerical simulations of the process. The theoretical solution also shows that the critical point $z_c^n$ does not diverge with the increasing generation *n*, but rather trends to a fixed value $z_c^{\infty }\approx 2.455$, at which $\psi _c^\infty \approx 0.512$. This means that the percolation transition becomes discontinuous when *n* → ∞.

To clarify the types of the percolation transition from simulations, we study the finite-size scaling of the order parameter $\psi ^n_c$ at the critical point (see Methods). Figure 2b shows that the simulation results of the first several generations have the same scaling for large *N*, which indicates that the order parameter $\psi ^n_c$ will vanish when *N* → ∞. This implies that these transitions are all continuous.

However, for a large *n*, the simulation results appear to deviate from the finite-size scaling of a continuous phase transition, see Fig. 2a. For a better understanding of this, we further show the distribution of $\psi ^n_c$ obtained in each individual realization. Figure  2c takes generation *n* = 10 as an example, see Section III of the Supplementary Information for other generations. The results show a heavy-tailed distribution instead of being confined to a small region as in classical percolation transition [25]. Especially when the system size is small (see the case *N* = 2^16^), a bimodal distribution similar to that of the discontinuous percolation transition can be found, namely, $\psi ^n_c$ around zero corresponds to the non-percolating realizations and the larger values for the percolating realizations. That is why we cannot observe a finite-size scaling in Fig. 2b for large *n*. In addition, Figure 2c also shows that the heavy tail will disappear with increasing system size. Thus, the similar scaling to that of the classical percolation is expected for very large systems.

For *n* = ∞, the distribution of $\psi ^n_c$ becomes a standard bimodal distribution (see Section III of the Supplementary Information), which allows us to identify the non-percolating realizations and remove them from the subsequent analysis. Then, the fitted result of the percolating realizations $\psi _{c0}^{\infty }\approx 0.514$ shown in Fig. 2d is in good agreement with the theoretical analysis, indicating a discontinuous percolation transition.

Moreover, for a finite system the infinite generation just corresponds to a generation *n*_*c*_, for which the late generations do not further alter the results. When *n* → *n*_*c*_, the system will demonstrate a much sharper percolation transition to that for the thermodynamic limit. However, *n*_*c*_ is varied for different network realizations, and the corresponding largest clusters are thus much different. As a result, the simulation results of the largest clusters at the theoretical threshold are distributed broadly. Consequently, the heavy-tailed distribution is found (see Fig. 2c), and the order parameters obtained by averaging over these become larger than the expectation of the finite-size scaling (see Fig. 2b). Note that *n*_*c*_ generally increases with the system size, thus the heavy-tailed distribution shown in Fig. 2c is more obvious for smaller systems.

As $p_c^{n-1}<p_c^{n}$, the percolation transition of generation *n* just corresponds to a classical percolation process (with a rescaled control parameter) in the supercritical phase of generation *n* − 1. This indicates that there is no essential difference between the percolation transition of two consecutive generations. As an immediate consequence, all the finite generations must belong to the same universality as the classical percolation, and the scaling behavior changes abruptly from the class shown in Fig. 2b to that of Fig. 2d when *n* → ∞.

**Figure 3.**
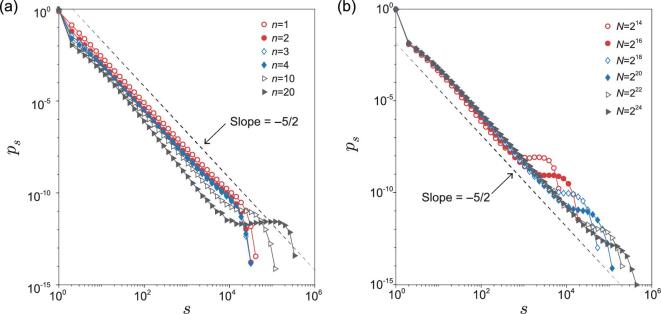
The cluster size distribution *p*_*s*_ at the critical point. (a) *p*_*s*_ for different generations *n*; the network size is *N* = 2^20^. (b) *p*_*s*_ of generation *n* = 10 for different network sizes *N*.

To further confirm the universality class of the history-dependent percolation model, we study the cluster size distribution *p*_*s*_. As pointed above, the largest clusters around the critical point are distributed broadly for a large *n*. A direct result of this is increasing the probability of finding large clusters in the system. For the cluster size distribution, this results in a cocked tail before a normal exponential cutoff, and becomes more and more apparent with increasing generation *n* (see Fig. 3a). However, from Fig. 3b, we find that this phenomenon weakens with increasing system size *N*, so a distribution similar to that of classical percolation can be expected for larger systems, that is a power-law with an exponential cutoff (without a cocked tail). This also suggests that the universality class is the same for all finite generations of the iterated percolation process.

### Network with SF layers

We now study the case where both layers are SF networks. In particular, we assume the power-law degree distribution [26]

(1) \begin{equation*} p_k =ck^{-\gamma },\quad k=m, m+1, \dots , K,\end{equation*}

where *c* is a normalization factor, and *m* and *K* are the lower and upper bounds of degree, respectively. If *K* is large enough and γ > 1, the normalization factor is approximately *c* ≈ (γ − 1)*m*^γ − 1^. In the simulation, networks are constructed by generating node degree values with equation (1) and then connecting the nodes with the configuration model. As the average degree is fixed by equation (1), we activate a fraction *p* of links of both network layers to control the effective mean degree and trigger the percolation process. In this section, we therefore seek the critical point in terms of critical probability *p*_*c*_, not critical average degree $z$_*c*_ as in the previous section.

It is known that when γ > 3, such SF network also has a non-trivial critical point similar to that of ER networks [3]. Consequently, the results are similar to those found for ER networks. Here we focus on the case γ ∈ (2, 3) which is realized in many real-world networks [27]. Previous studies have demonstrated that the standard percolation in this case has zero critical point [4,5].

**Figure 4.**
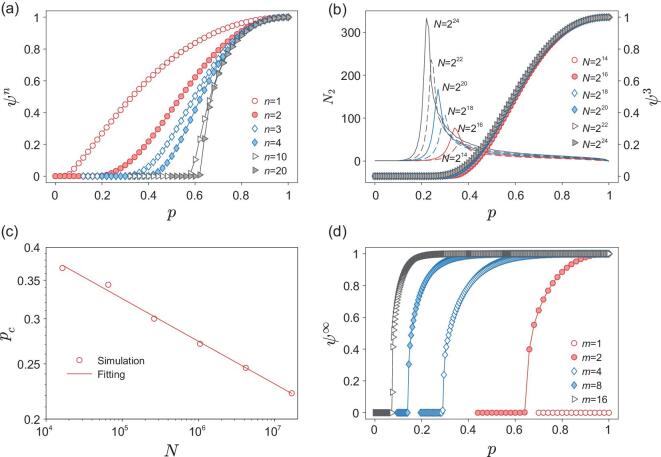
The simulation results for SF networks with the degree distribution given by equation (1) where *m* = 2, $K=\sqrt{N}$ and γ = 2.5. (a) The size of the giant cluster ψ^*n*^ as a function of probability *p* for different generations. The network size is *N* = 2^16^. (b) The size of the giant cluster ψ^*n*^ of generation *n* = 3 as a function of probability *p* for different network sizes *N*. The solid lines are the number of nodes in the second largest cluster *N*_2_. (c) The finite-size scaling of the pseudo-critical points for generation *n* = 3. The fitting curve takes the form *p*_*c*_ ∝ *N*^−α^ with α = 0.075 ± 0.003. (d) The size of the giant cluster ψ^*n*^ as a function of probability *p* for infinite generations with different minimum degrees *m*. The network size is *N* = 2^16^.

The simulation results shown in Fig. 4a show that the percolation transition becomes sharper and sharper as the generation increases. However, Fig. 4b demonstrates that the pseudo-critical point indicated by the maximum of the second largest cluster decreases with the system size, which suggests a vanished critical point can also be found for infinite system. From the theoretical analysis (see Methods and the Supplementary Information), we found that all the finite generations on such networks have zero critical point as the classical percolation, which is further confirmed by the finite-size scaling of pseudo-critical point shown in Fig. 4c.

We also find that to observe a non-trivial critical point on such networks, it requires endless iterations, large *m* and broad degree distribution, otherwise the infinite generation will destroy the whole network (see Section I-C in the Supplementary Information for details). Generally speaking, SF networks with more connections and border degree distributions are more likely to survive in the infinite iterated processes. Figure 4d demonstrates that when *m* > 1, a discontinuous percolation transition with non-trivial critical point can also be found for SF networks.

### Real multiplex networks

Our brain is a complex system and network neuroscience holds great promise for expanding our understanding of a healthy brain functioning, brain diseases, brain development and brain aging [28,29]. Simply, the brain can be modeled by a network, where the brain regions and their connections constitute the set of nodes and the set of links, respectively. Here, we consider the human brain networks which are constructed using the high-resolution brain atlas with 1024 Regions-of-Interest (ROIs) [30], that is the network thus has 1024 nodes. This multiplex network has two layers, which capture functional correlations and morphological similarity of the human brain based on ROIs, respectively. For the functional brain layer, the mean time series is extracted for each ROI by averaging the time series of all voxels (small measured volumes in three-dimensional space) within it. Then, we calculate the Pearson correlation for each pair of ROIs and generate a 1024 × 1024 correlation matrix. For the morphological brain layer, we estimate the interregional similarity in the distribution of regional gray matter volume in terms of the Kullback-Leibler divergence measure [31]. By this construction, both layers are represented by weighted complete networks. We tune the resulting networks by choosing the average degree in each layer, $z$, and thus obtain the corresponding unweighted networks where only links with the highest weights are kept. See the last section of the Supplementary Information for a detailed description of the data and the MRI data preprocessing strategy.

**Figure 5.**
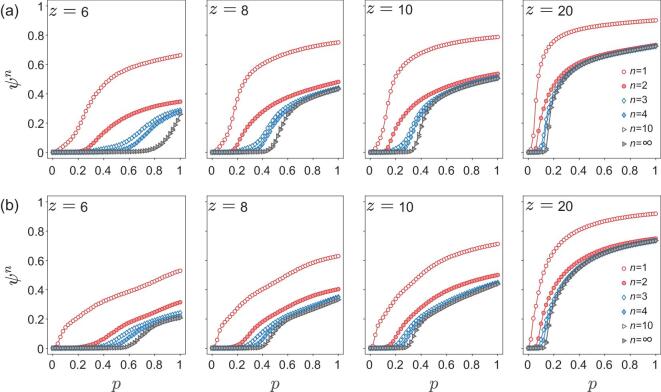
History-dependent percolation on human functional-morphological brain networks for different average degree values ($z$); *p* is the link occupation probability; ψ^*n*^ is the size of the giant cluster given the percolation generation *n*. (a) The HC participant’s bilayer brain network. (b) The MDD participant’s bilayer brain network.

In Fig. 5, we compare the results of our percolation model on two different human functional-morphological brain networks, one from a major depressive disorder (MDD) participant and one from a healthy control (HC) participant. For the MDD data, both the critical point and the giant cluster for a given *p* are smaller than that of HC, indicating that the brain network of the MDD participant is more vulnerable. For the infinite generation of the model, the pattern of the remaining nodes and links (the giant cluster) of the MDD data is sparser and more dispersed. These nodes are mainly located in frontal, parietal and occipital lobes, and do not form obvious community structures, whereas, for the HC participant, these nodes are close together and are mainly located in the highly myelinated brain regions (i.e. the motor-somatosensory strip in the central sulcus, the visual cortex in the occipital lobe). This suggests that for the HC participant, the giant cluster identified by a finite-generation percolation process may reflect the biological meanings. See Fig. 7 in the Supplementary Information for details.

**Figure 6.**
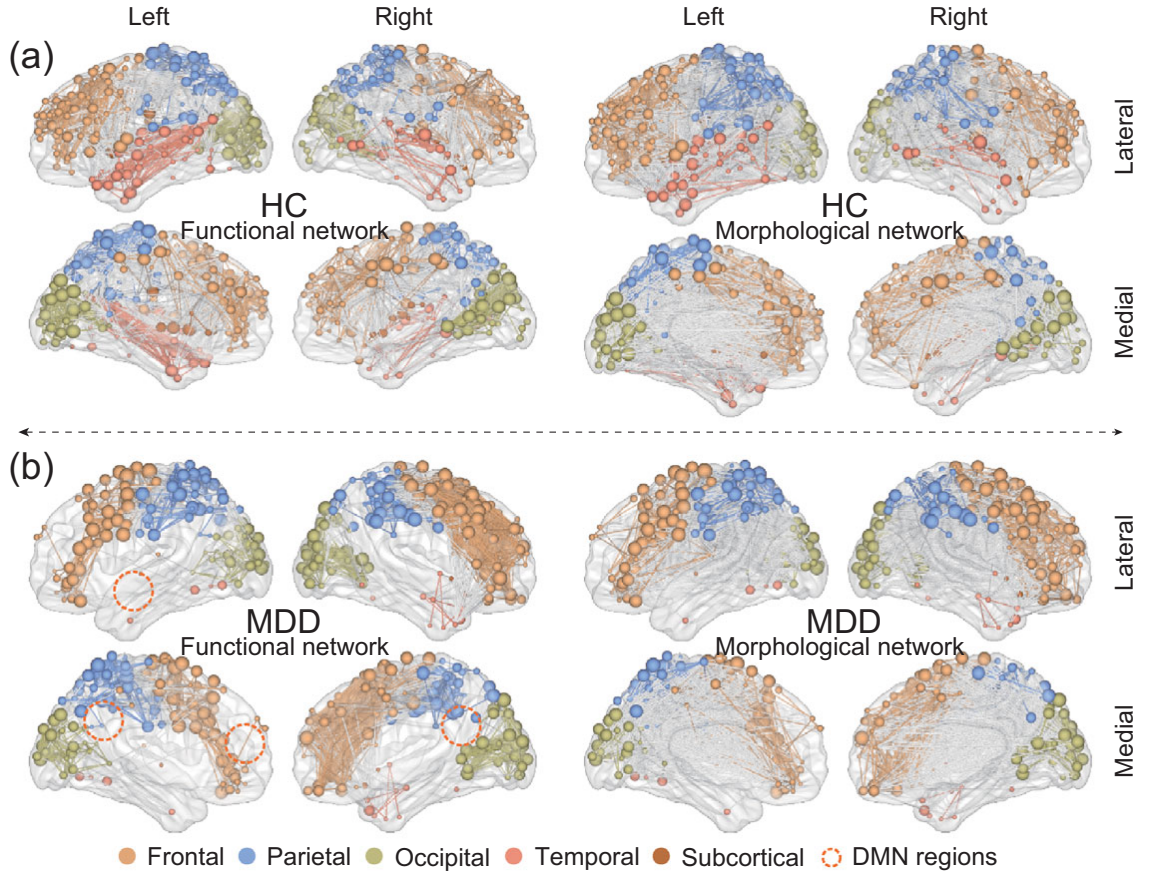
Visualization of human brain bilayer networks at degree $z$ = 20 and 1024 parcellation templates when *p* is slightly larger than the corresponding *p*_*c*_. (a) The HC’s bilayer brain network. (b) The MDD’s bilayer brain network. We show the lateral and medial brain of each hemisphere. The visualization is done with BrainNet viewer [32].

When we increase the average degree to $z$ = 20, both brain networks become robust, and the percolation transition of infinite generation becomes sharper (see Fig. 5). As shown in Fig. 6, for the HC participant, the giant cluster of the infinite generation diffuses across the frontal, parietal, occipital, temporal and subcortical lobes. Compared with HC data, the MDD network shows an apparent deficiency of nodes in frontal, occipital and temporal lobes, some of which belong to the default mode network (DMN) [33]. These DMN regions play an important role in our advanced cognitive abilities, such as executive control, visual and auditory sense, and some pieces of evidence support the decreased functional connectivity [34,35] and frontal cortical thinning [36] in these regions for MDD participants, see Fig. 6. Therefore, our model opens a new avenue toward detecting abnormal nodes or components between healthy participants and those with disease from a comprehensive functional-morphological perspective, which can help us detect the potential connectome-based MRI biomarker and gain new insights into the mechanisms of some brain disorders. Importantly, these findings are beyond the single modal imaging/layer and traditional percolation, thus providing a novel understanding and convergent results for MDD. Furthermore, our model is easy to expand to the networks with three or more layers and could be used to investigate the similarities, differences and comprehensive understanding of various brain disorders. Note that the current study is a single case validation. Besides, our brains are highly personalized and a large sample size is needed to infer robust conclusions [37,38]. Further study should use larger sample sizes or different brain disorders to verify our model.

We also applied our model to a bilayer social network composed of users who are active on both Twitter and FriendFeed [39]. Among the 150 684 common users of the two networks, there are 8 308 326 and 5 270 665 links in the Twitter and FriendFeed layers, respectively. The results can be found in Fig. 6 in the Supplementary Information. We find that the percolation transition occurs at much lower activation probability *p* than that of the brain network data. This is a direct consequence of the average degree in the social network ($z$ ≈ 110 and 70 in the two network layers, respectively) being substantially higher than in the brain network ($z$ = 6, 8, 10, 20). The Twitter-FriendFeed network reaches the steady state after four generations, and the discontinuous percolation transition is also absent. This can be a result of many links (about $37\%$ of the total number) that occur in both network layers, which render subsequent process generations equivalent to the first two or three generations of the case discussed in Fig. 4.

These results suggest that to observe non-trivial behaviors in iterative percolation, one needs to study multiplex networks with limited layer overlap. To fully understand the relation between the layer overlap and the dynamics of the iterative percolation remains a future challenge. The human brain network reaches the steady state after 10 generations. At this point, the percolation transition is more abrupt than that for smaller iteration values. With respect to the small size of the studied system (1024 nodes), the possibility that a larger brain network (achievable with a higher imaging resolution) would display yet more abrupt transition, and thus suggest a discontinuous percolation transition in the thermodynamic limit, remains open. However, whether the percolation transition is discontinuous or not, the process provides a series of methods to analyse network structures.

## DISCUSSION

In summary, we introduce a history-dependent percolation model to study the critical behavior of the percolation transition on multiplex networks. The percolation process is run iteratively on different layers. Instead of focusing solely on the steady state, we pay more attention to finite generations, each of which can be considered as an independent model. For example, *n* = 1 corresponds to the common model of information or disease spreading on monopartite networks; *n* = 2 can be used to describe the interplay between the spreading of disease and immunity information [20]; *n* = 3 can be used to model the information spreading on multiplex social networks where users communicate through multiple channels. The general example of multilayer propagation is the one where online communication influences user offline behavior and then couples back to online communication. A typical example of *n* = ∞ is the study of cascading failures in coupled networks [12]. In this sense, our model provides a unified framework to study the percolation process on multiplex networks.

We investigate both ER networks and SF networks with power-law exponent 2 < γ < 3. The results reveal that the intermediate state of the recursive process can be also used to uncover meaningful structures, and therefore the percolation transition should be defined and studied in each generation. For any finite generations, the percolation transition on random networks shows a continuous transition and belongs to the same universality class, while SF networks have the critical point trending to zero as the network size grows. When *n* = ∞, a discontinuous transition exists for both networks [12]. In essence, this is because the percolation transition of generation *n* emerges from the supercritical phase of the percolation of generation *n* − 1. As a result, it inherently cannot generate a new universality class [15]. However, when *n* diverges, *n* − 1 is also divergent, this relation of the critical state is broken. Then, the new phenomenon, that is discontinuous transition, emerges.

Furthermore, on one hand, our result indicates that the continuous transition found in real systems could be the result of a combination of many sequential processes, see an example in Ref. [20]. On the other hand, to observe the abrupt percolation transitions in real multiplex networks, for which the generation cannot go to infinite actually, the model may need to be extended by, for example, considering the space embedding of network layers [40], modeling relative importance of inter- and intra-layer connections [41] or introducing cores of ‘high quality’ edges [42]. Our model can be easily extended to a general case with more layers. This would make it possible to use the generalized model to analyse the percolation transition on a temporal network comprising several layers corresponding to different time points [43].

Beyond the theoretical analysis, we showed that the outcomes of the iterative percolation process can be used to characterize real networks whose percolation properties differ markedly between systems (such as the used brain scan and social network data) as well as between various samples of networks from the same class (such as the brain scan data of a healthy participant and a participant with a mental disorder). The proposed model can thus become a useful tool for evaluating and, more importantly, comparing structural properties of multiplex networks. The model represents an important step towards understanding the history-dependent dynamic processes on multiplex networks, and may prove useful in important practical applications like link prediction [44], vital node identification [45] and community detection [46] in multiplex networks.

## METHODS

### Mean-field theory analysis

To obtain the exact analytical solution of the critical point, we consider the giant cluster in the infinite system [12]. We introduce first the function $\mathfrak {F}(x)$ which returns the size of the giant cluster of a network ensemble with given degree distribution, when a fraction *x* of nodes is chosen at random and used to construct the giant cluster. Note that the fraction obtained by function $\mathfrak {F}(x)$ is with respect to the actually used nodes. The size of the giant cluster with respect to the original network is thus $x\mathfrak {F}(x)$.

Leaving the specific form of $\mathfrak {F}(x)$ aside, which may be a group of equations or a dataset obtained by Monte Carlo simulations, we now lay out the general analytical framework for the history-dependent percolation process. As the network’s layers are in general different, we assume that layers *A* and *B* have functions $\mathfrak {F}_A(x)$ and $\mathfrak {F}_B(x)$, respectively. In addition, we label the size of the giant cluster in generation *n* as ψ^*n*^, and the fraction of the nodes that can be used to construct the giant cluster in generation *n* as *S*^*n* − 1^. The function $\mathfrak {F}(x)$ allows us to write

(2) \begin{equation*} \psi ^n=S^{n-1}\mathfrak {F}(S^{n-1}), \end{equation*}

where $\mathfrak {F}(x)$ is $\mathfrak {F}_A(x)$ and $\mathfrak {F}_B(x)$ for odd and even generations, respectively. All we need to do now is to find *S*^*n* − 1^ for each generation *n*.

For an infinite system, the giant cluster of generation *n* can only emerge from the giant cluster of generation *n* − 1. So, leveraging this recursive relationship, the fraction of nodes that can be used to construct the giant cluster *S*^*n*^ for an odd *n* satisfies

(3) \begin{equation*} S^{n} = S^0\mathfrak {F}_A(S^{n-1}), \end{equation*}

and for an even *n*,

(4) \begin{equation*} S^{n} = S^0\mathfrak {F}_B(S^{n-1}). \end{equation*}

In addition, if one removes a fraction 1 − *p* of nodes in the initial configuration to trigger the iterated percolation, then *S*^0^ = *p*. If the removal is for links, such as the ones used in Figs 4 and 5, the function $\mathfrak {F}(x)$ for the diluted network should be replaced with $\mathfrak {F}(px)$ as the degree distribution has changed [8].

If functions $\mathfrak {F}_{A,B}(x)$ are known, equations (2)–(4) can be used to calculate the size of the giant cluster for any generation and also the critical point. For ER networks $\mathfrak {F}(x)$ can be obtained by solving a self-consistent equation; however, there is no closed form for SF networks and the theoretical analysis can be done only around the critical point, see Section I of the Supplementary Information for details.

If function $\mathfrak {F}(x)$ has a non-trivial critical point *x*_*c*_ below which $\mathfrak {F}(x)=0$ and $\mathfrak {F}(x_c)=0$, such as ER networks, equations (3) and (4) show that the critical point of generation *n* corresponds to a non-zero *S*^*n* − 1^, that is the supercritical state of generation *n* − 1. This indicates that there is no essential difference between the percolation transition of the two generations, and consequently the first generation and also all finite generations of iterative percolation demonstrate a continuous percolation transition. Figure 2a takes ER networks as the example to show that the resulting ψ^*n*^ agrees well with numerical simulations of the process.

The recursive relations equations (3) and (4) have their fixed point $S=\mathfrak {F}(S)$, which corresponds to the infinite generation, and allows us to find the fixed point for ER layers $z_c^{\infty }\approx 2.455$ and $S_c=\left(1+\sqrt{1-z_c^\infty /2}\right)/2\approx 0.715$, see Section I-C of the Supplementary Information for details. The critical order parameter that corresponds to the found *S*_*c*_ is $\psi _c^\infty =(S_c)^2\approx 0.512$, which also agrees well with numerical simulations shown in Fig. 2d. The giant cluster size thus undergoes a discontinuous phase transition at $z_c^{\infty }$.

For SF networks, the recursive relations equations (3) and (4) can also be used to obtain the critical point of each generation. As $\mathfrak {F}(px)$ has a zero critical point for 2 < γ < 3, we can find that all the finite generations give a vanished critical point *p*_*c*_ = 0 by using equations (3) and (4), recursively (see Section I-B of the Supplementary Information for details). By examining the fixed point of equations (3) and (4), we can also find that the infinite generation demonstrates a discontinuous percolation transition, see Section I-C of the Supplementary Information for details.

### The order parameter at the critical point

As a topological phase transition, there is no free energy that can be used to determine the type of the percolation transition. Therefore, the transition with a step-like changing of the order parameter at the critical point is often treated as a discontinuous percolation transition [2,12,16,24,41,42], and the continuous percolation transition is for that with continuous changing at the critical point. That is to say, the discontinuous percolation transition has a non-zero order parameter at the critical point, and a zero order parameter can be found for continuous percolation transition.

However, because of finite sizes of the systems used in the simulation, both types of percolation transitions could give a non-zero order parameter at the critical point. In this way, we use the finite-size scaling to check the types of percolation transition in simulations. For a continuous transition, the order parameter ψ_*c*_ must decrease with increasing system size and becomes zero for an infinite system, which corresponds to ψ_*c*_ ∝ *N*^−ε^ where ε = 1/3 for random networks [25]. For a discontinuous transition, the order parameter ψ_*c*_ takes a bimodal distribution, the values around zero correspond to the non-percolating realizations and the larger values for the percolating realizations. Excluding the non-percolating realizations, the finite-size scaling thus takes the form ψ_*c*_ ∼ ψ_*c*0_ + *O*(*N*^−ϵ^), where ψ_*c*0_ is the order parameter at the critical point for an infinite system. Therefore, Fig. 2b–d just indicate that all the finite generations take the continuous percolation transition, and the infinite generation takes the discontinuous percolation transition. However, because of finite-size effects, larger systems are needed to observe a better finite-size scaling.

## Supplementary Material

nwaa029_Supplemental_File
