# Supplementary material for: History-dependent percolation on multiplex networks
Source: Natl Sci Rev. 2020 Feb 20;7(8):1296–305. doi: 10.1093/nsr/nwaa029 (PMC8288926; doi:10.1093/nsr/nwaa029)
Supplement: nwaa029_Supplemental_File [file nwaa029_supplemental_file.pdf]

Supplementary information for "History-dependent percolation on multiplex networks"

Ming Li,[1] Linyuan Lü,[2,3,4,*] Youjin Deng,[5] Mao-Bin Hu,[1]
Hao Wang,[2] Matúš Medo,[2] and H. Eugene Stanley[3,6]

[1]*Department of Thermal Science and Energy Engineering, University of Science and Technology of China, Hefei 230026, P. R. China*

[2]*Institute of Fundamental and Frontier Sciences, University of Electronic Science and Technology of China, Chengdu 610054, P. R. China*

[3]*Alibaba Research Center for Complexity Sciences, Hangzhou Normal University, Hangzhou 310036, P. R. China*

[4]*Beijing Computational Science Research Center, Beijing 100193, P. R. China*

[5]*Hefei National Laboratory for Physical Sciences at Microscale, Department of Modern Physics, and CAS Center for Excellence and Synergetic Innovation Center in Quantum Information and Quantum Physics, University of Science and Technology of China, Hefei 230026, P. R. China*

[6]*Department of Physics and Center for Polymer Studies, Boston University, Boston, Massachusetts 02215, USA*

(Dated: February 15, 2020)

CONTENTS

I. Theory 3

A. General formalism 3

1. $M = 2$ 3

2. $M = 3$ 4

B. The critical point 6

1. Two-layer Erdős–Rényi (ER) networks 6

2. Two-layer scale-free (SF) networks 7

C. Infinite generation 8

1. Two-layer ER networks 9

2. Two-layer SF networks 10

II. Algorithm 11

III. The largest cluster at the critical points 13

IV. Social network 14

V. Brain network 14

VI. MRI data and processing 16

References 16

* linyuan.lv@uestc.edu.cn

I. THEORY

A. General formalism

We introduce the function $\mathfrak{F}(x)$ to represent the size of the giant cluster of a network ensemble with a given degree distribution, when a fraction x of nodes are chosen at random and used to construct the giant cluster. Note that the fraction obtained by function $\mathfrak{F}(x)$ is with respect to the number of actually used nodes (fraction x of all nodes). The size of the giant cluster with respect to the original network is thus $x\mathfrak{F}(x)$.

For our model, different layers can have different degree distributions, the above-described function then differs from one layer to another, and we can label it $\mathfrak{F}_i(x)$ for layer i. Assuming the fraction of the nodes that can be used to construct the giant cluster in generation n is S^{n-1}, then the function $\mathfrak{F}(x)$ allows us to write the size of the giant cluster in generation n as

$$\psi^n = S^{n-1}\mathfrak{F}_{l_n}(S^{n-1}), \tag{1}$$

where l_n is the layer used in generation n.

In the infinite system, a giant cluster can only emerge from the giant cluster of the prior generation. Consequently, there is a recursive relation between the fraction S^n of two successive generations, which can also be expressed by function $\mathfrak{F}(x)$,

$$S^{n-1} = S^0 \prod_{i=(n-M)H(n-M)+1}^{n-1} \mathfrak{F}_{l_i}(S^{i-1}), \quad n \geq 2. \tag{2}$$

Here, M is the number of layers, and $H(x)$ is the Heaviside step function, *i.e.*,

$$H(x) = \begin{cases} 1, & x \geq 0, \\ 0, & x < 0. \end{cases} \tag{3}$$

Next, we take $M = 2, 3$ as examples to provide an interpretation of Eq. (2).

1. $M = 2$

The case $M = 2$ is the one shown in the main text, so we use the same notation, *i.e.*, the two layers are labeled A and B. In generation $n = 1$, all nodes can be used to construct the giant cluster, *i.e.*, $S^0 = 1$. The giant cluster size ψ^1 can be thus expressed as $\psi^1 = S^0\mathfrak{F}_A(S^0)$. The giant cluster

of generation 2 emerges from the giant cluster of generation $n = 1$. The fraction of nodes $S^1 = \psi^1$ is therefore the starting point for generation $n = 2$ and we can write $\psi^2 = S^1 \mathfrak{F}_B(S^1)$.

For generation $n = 3$, some nodes in the giant cluster of generation $n = 1$ can no longer be used to construct the giant cluster, and the fraction is $\psi^1 - \psi^2 = S^1[1 - \mathfrak{F}_B(S^1)]$. Removing these nodes from the giant cluster (fraction $S^1 = \psi^1$) is equivalent to removing the same fraction of nodes from S^0, since the other nodes (fraction $1 - S^1$) that are not used to construct the giant cluster of generation $n = 1$ do not belong to S^1 and ψ^2. So the total fraction of nodes can not be used in generation $n = 3$ is $S^0[1 - \mathfrak{F}_B(S^1)]$. In this way, generation $n = 3$ is equivalent to randomly using a fraction $S^2 = S^0 \mathfrak{F}_B(S^1)$ of nodes to construct the giant cluster. It is easy to see that the general formula for the fraction of nodes that can be used to construct the giant cluster S^n for an odd n is

$$S^n = S^0 \mathfrak{F}_A(S^{n-1}), \tag{4}$$

and for an even n,

$$S^n = S^0 \mathfrak{F}_B(S^{n-1}). \tag{5}$$

These two equations are clearly a particular form of Eq. (2).

2. $M = 3$

We further take $M = 3$ as another example. In generation $n = 1$, all nodes can be used to construct the giant cluster, *i.e.*, $S^0 = 1$. The giant cluster size ψ^1 can be thus expressed as $\psi^1 = S^0 \mathfrak{F}_1(S^0)$. The fraction of nodes $S^1 = \psi^1 = S^0 \mathfrak{F}_1(S^0)$ is therefore the starting point for generation $n = 2$ and we can write $\psi^2 = S^1 \mathfrak{F}_2(S^1)$. Similarly, for generation $n = 3$, the fraction $S^0 - \psi^2$ of nodes cannot be used to construct the giant cluster. That is $S^2 = \psi^2 = S^1 \mathfrak{F}_2(S^1) = S^0 \mathfrak{F}_1(S^0) \mathfrak{F}_2(S^1)$, and $\psi^3 = S^2 \mathfrak{F}_3(S^2)$.

For generation $n = 4$, we will reuse layer 1 to construct the giant cluster, however, some nodes in the giant cluster of generation $n = 1$ can no longer be used to construct the giant cluster, and the fraction is $\psi^1 - \psi^3 = S^1[1 - \mathfrak{F}_2(S^1)\mathfrak{F}_3(S^2)]$. Removing these nodes from the giant cluster (fraction $S^1 = \psi^1$) is equivalent to removing the same fraction of nodes from S^0, since the other nodes (fraction $1 - S^1$) that are not used to construct the giant cluster of generation $n = 1$ do not belong to S^1 and ψ^3. So the total fraction of nodes can not be used in generation $n = 4$ is $S^0[1 - \mathfrak{F}_2(S^1)\mathfrak{F}_3(S^2)]$. In this way, generation $n = 4$ is equivalent to randomly using a fraction $S^3 = S^0 \mathfrak{F}_2(S^1)\mathfrak{F}_3(S^2)$ of nodes to construct the giant cluster. By analogy, the fraction of nodes

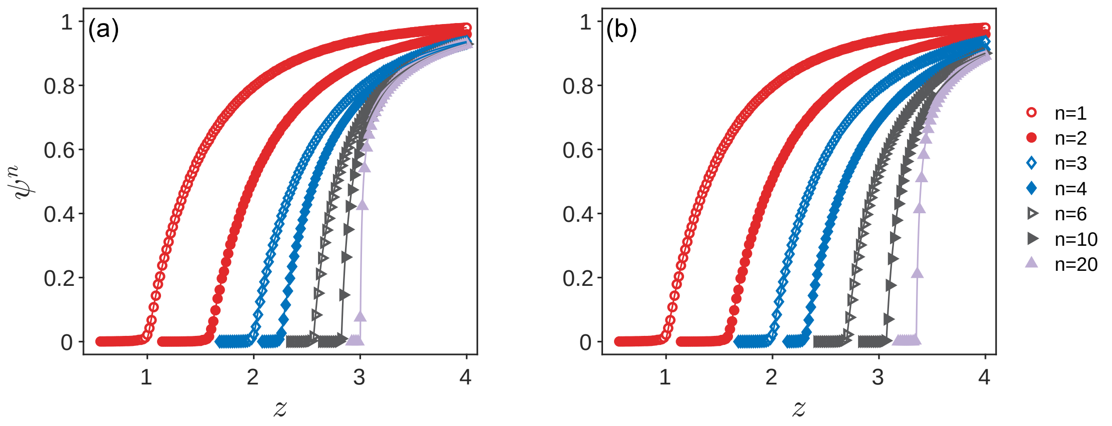

FIG. 1. The size of the giant cluster ψ^n as a function of the average degree z for different generations n. In the simulation, the system size is $N = 2^{16}$, and all layers are ER networks with the same average degree z. The solid lines are the theoretical results obtained by Eqs. (1) and (2), numerically. (a) $M = 3$. (b) $M = 4$.

that can be used to construct the giant cluster can be represented as

$$S^1 = S^0 \mathfrak{F}_1(S^0), \tag{6}$$

$$S^2 = S^0 \mathfrak{F}_1(S^0)\mathfrak{F}_2(S^1), \tag{7}$$

$$S^3 = S^0 \mathfrak{F}_2(S^1)\mathfrak{F}_3(S^2), \tag{8}$$

$$S^4 = S^0 \mathfrak{F}_3(S^2)\mathfrak{F}_1(S^3), \tag{9}$$

$$S^5 = S^0 \mathfrak{F}_1(S^3)\mathfrak{F}_2(S^4), \tag{10}$$

$$\vdots$$

$$S^n = S^0 \mathfrak{F}_{l_{n-2}}(S^{n-2})\mathfrak{F}_{l_{n-1}}(S^{n-1}). \tag{11}$$

A more general expression of these equations is just Eq. (2). In addition, if one removes a fraction $1 - p$ of nodes in the initial configuration to trigger the iterated percolation, then $S^0 = p$. If the removal is for links, the function $\mathfrak{F}(x)$ for the reduced network should be replaced with $\mathfrak{F}(px)$.

If functions $\mathfrak{F}(x)$ of all layers are known, we can get the theoretical results by Eqs. (1) and (2), analytically or numerically. In Fig. 1, we give the simulation results for $M = 2$ and 3, which are in agreement with the theory well.

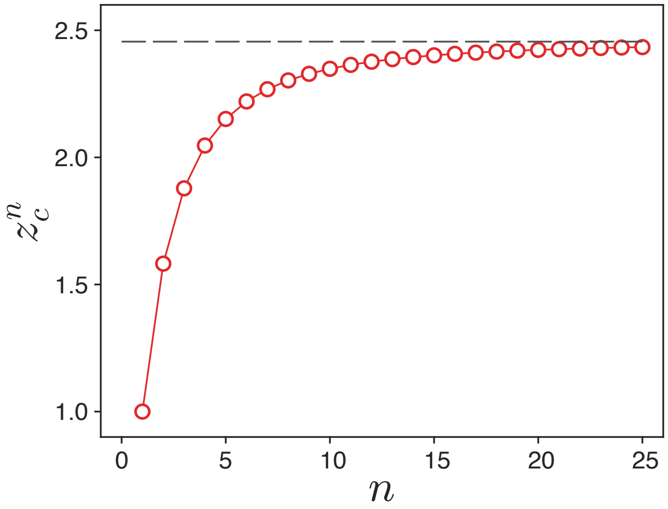

FIG. 2. The critical point z_c^n of ER networks obtained by our method for different generations n. The dotted line is the critical point for the infinite generation, $z_c^\infty \approx 2.455$.

B. The critical point

The critical point of this system is determined by function $\mathfrak{F}(x)$ in Eq. (1). Assuming function $\mathfrak{F}(x)$ has a critical point x_c below which $\mathfrak{F}(x) = 0$. Thus, substituting $S^{n-1} = x_c$ into Eqs. (1) and (2), we can obtain the critical point of for any generation. Next, we take the case used in the main text to show the details, that is a two-layer network with the same degree distribution in each layer.

1. Two-layer Erdős–Rényi (ER) networks

For ER networks, function $\mathfrak{F}(x)$ satisfies the self-consistent equation

$$\mathfrak{F}(x) = 1 - e^{-zx\mathfrak{F}(x)}, \tag{12}$$

with the critical point $x_c = 1/z$ below which $\mathfrak{F}(x) = 0$. According to Eq. (1), the critical point of generation n corresponds to $S^{n-1} = x_c = 1/z$. For $S^0 = 1$ and $M = 2$, the iterative Eq. (2) simplifies to the form $S^n = \mathfrak{F}(S^{n-1})$. The critical point of generation n can thus be further developed as $S^{n-2} = \mathfrak{F}^{-1}(S^{n-1}) = \mathfrak{F}^{-1}(1/z)$, where $\mathfrak{F}^{-1}(x)$ is the inverse function of $\mathfrak{F}(x)$ which reads

$$\mathfrak{F}^{-1}(x) = -\frac{\ln(1-x)}{zx} \tag{13}$$

and one can easily verify that $\mathfrak{F}[\mathfrak{F}^{-1}(x)] = x$. One can continue the development with S^{n-3}, S^{n-4}, and so on until

$$\underbrace{\mathfrak{F}^{-1}\left(\cdots\mathfrak{F}^{-1}\left(\mathfrak{F}^{-1}(1/z_c^n)\right)\right)}_{n-1\text{ times}} = S^0 = 1. \tag{14}$$

For any generation n, we can use this equation to get the critical point z_c^n, numerically. The theoretical solution also shows that the critical point z_c^n does not diverge with the increasing of generation n), but rather trends to a fixed value $z_c^\infty \approx 2.455$ (see Fig.2).

Furthermore, one also can note that $\mathfrak{F}(x) \to 0$ when $x \to x_c^+$, indicating that the first generation and consequently also all finite generations of iterative percolation demonstrate a continuous percolation transition, *i.e.*, $\psi_c^n = 0$.

2. *Two-layer scale-free (SF) networks*

For this case, we assume that both the two layers have the same power-law degree distribution

$$p_k = ck^{-\gamma}, \qquad k = m, m+1, \ldots, K, \tag{15}$$

where c is the normalization factor, and m and K are the lower and upper bounds of degree, respectively. If K is large enough and $\gamma > 1$, the normalization factor is approximately $c \approx (\gamma-1)m^{\gamma-1}$.

Since the average degree is fixed by Eq. (15), we randomly remove a fraction $1-p$ of links of both network layers to control the effective mean degree. Then, the function $\mathfrak{F}(px)$ for this system can be expressed by the generating functions

$$R = 1 - G_1(1 - pRx), \tag{16}$$

$$\mathfrak{F}(px) = 1 - G_0(1 - pRx), \tag{17}$$

where R is an auxiliary variable, $G_0(x) = \sum_k p_k x^k$ and $G_1(x) = \sum_k p_k k x^{k-1}/z$ are the generating functions of the degree and excess-degree distributions, respectively. From Eqs. (16) and (17), the critical point of $\mathfrak{F}(px)$ can be obtained

$$p_c = \frac{1}{G_1'(1)}, \tag{18}$$

below which $\mathfrak{F}(px) = 0$. Note that for $m = 1$, this formula gives a percolation threshold p_c larger than 1 when $\lambda > 3.47875\ldots$, indicating there is no percolation transition. This is due to the absent of the spanning cluster in such SF networks[1].

For $\gamma \in (2,3)$ which is realized in many real-world networks, $G_1'(1) \to \infty$ with the increasing system size. So function $\mathfrak{F}(px)$ gives a vanished critical point $p_c = 0$ with $\mathfrak{F}(p_c x) = 0$. According to Eq. (1), the critical point of generation n corresponds to $S^{n-1} = 0$. Then, using the iterative relation $S^n = \mathfrak{F}(pS^{n-1})$, it is clear that for any finite generation, the system gives a vanished critical point.

In addition, when $\gamma > 3$, function $\mathfrak{F}(px)$ has a non-trivial critical point as that of ER networks. Consequently, the results are similar to that we introduced above for ER layers, *i.e.*, there is a non-trivial percolation transition for any finite generation. Note that due to the strong heterogeneity of SF networks, some special critical exponents dependent on the exponent γ can be found [2, 3].

C. Infinite generation

For infinite iterations, layers are favored rather than generations. For convenience, we use subscript to differentiate layers instead of generations, such as ψ_i for the size of the giant cluster of layer i, and S_i for the fraction of nodes can be used to construct the giant cluster of layer i. Based on these notations, Eq.(2) can be rewritten as

$$S_i = S^0 \prod_{j \neq i}^{M} \mathfrak{F}_j(S_j). \tag{19}$$

Substituting this relation into Eq. (1), we have

$$\psi_i = S_i \mathfrak{F}_i(S_i) = S^0 \prod_{j=1}^{M} \mathfrak{F}_j(S_j) \equiv \psi. \tag{20}$$

This means that all the giant clusters (in different layers) have the same size. According to Eq. (20), the case of the infinite generation can be solved by the following equations

$$\psi = S^0 \prod_{i=1}^{M} \mathfrak{F}_i(S_i), \tag{21}$$

$$S_i = \frac{\psi}{\mathfrak{F}_i(S_i)}, \quad i = 1, 2, \ldots, M. \tag{22}$$

This form recovers the finding of the percolation on tree-like network of networks [4].

For a given function $\mathfrak{F}(x)$, we can solve Eqs. (21) and (22) to get the giant cluster and the critical point. To show how to analysis the infinite generation, we also take the case used in the main text as an example, *i.e.*, a two-layer network with the same degree distribution in each layer.

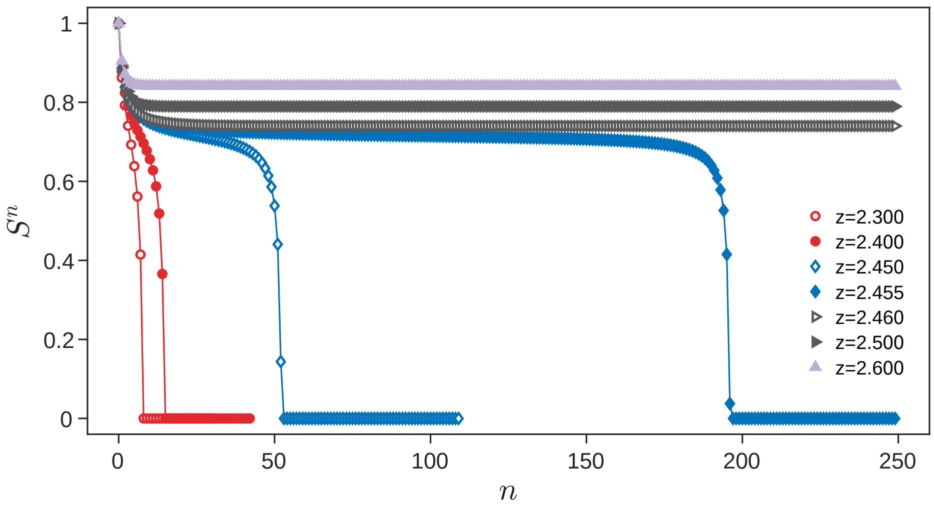

FIG. 3. The fixed points for Eqs. (4) and (5). The curves are the numerical solutions of two-layer ER networks, for which the two layers have the same $\mathfrak{F}(x)$ Eq. (12).

1. *Two-layer ER networks*

Since the two layers have the same degree distribution, Eqs.(21) and (22) reduce to

$$\psi = S^2, \tag{23}$$

$$S = \mathfrak{F}(S). \tag{24}$$

Here, $S^0 = 1$ is used. It is easy to know that the critical point is determined by Eq. (24). Together with Eq. (12), we have

$$S = 1 - e^{-zS^2}. \tag{25}$$

Below a critical point $z_c^\infty \approx 2.455$, this equation has only the trivial solution $S = 0$ because the "inverted Bell curve" on the right hand side (rhs) is under the linear left hand side (lhs) for any $S > 0$. At the critical point z_c^∞, the lhs and rhs touch in one point. Denoting the rhs as $f(S)$, the touching point S_c satisfies $f(S_c) = S_c$ and $f'(S_c) = 1$, which allows us to find its form

$$S_c = \frac{1}{2}\left(1 + \sqrt{1 - \frac{2}{z_c^\infty}}\right) \approx 0.7153. \tag{26}$$

The other existing solution $S_c = (1 - \sqrt{1 - 2/z_c^\infty})/2 \approx 0.2847$, is smaller than the corresponding critical point $x_c = 1/z_c^\infty \approx 0.4073$ of function $\mathfrak{F}(x)$. Further iterations will thus turn it to 0, which indicates that this smaller solution is not a real fixed point. The critical order parameter

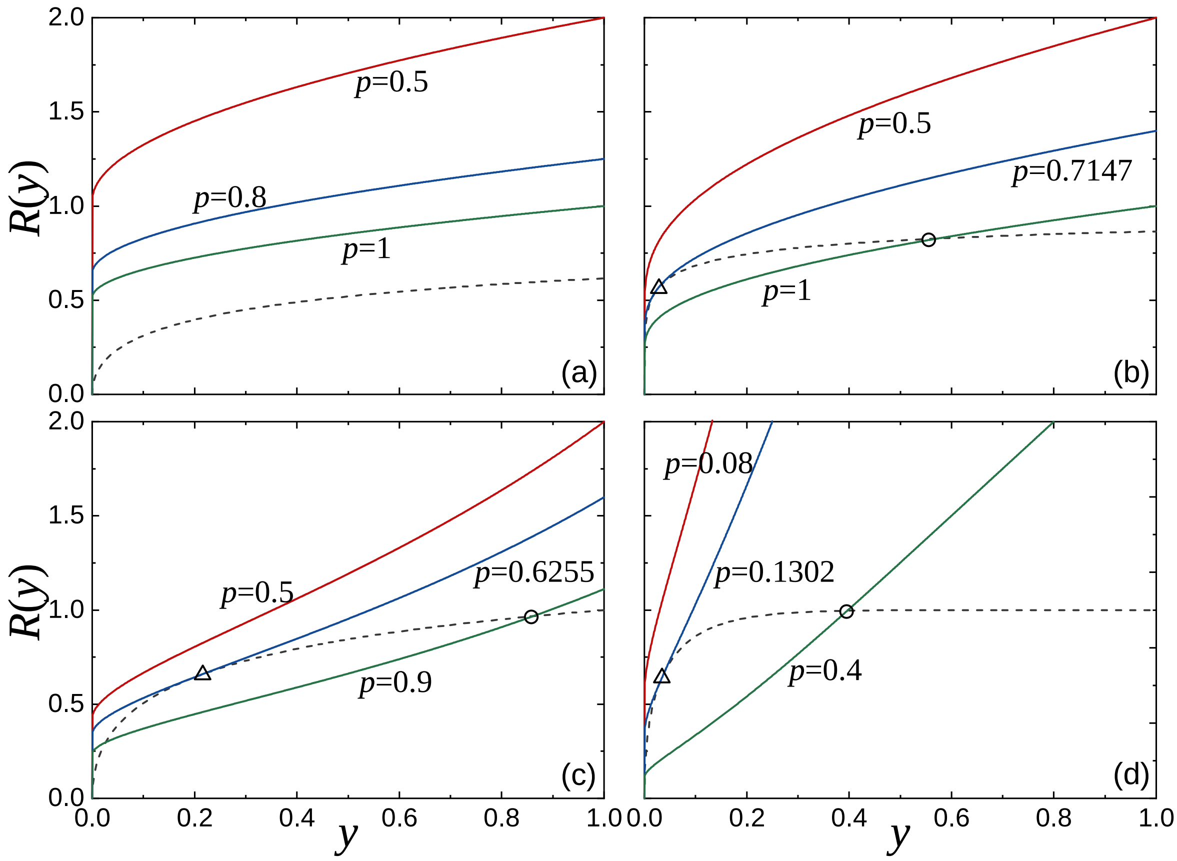

FIG. 4. A graphical representation of the numerical solution of Eqs. (27) and (28). To obtain these curves, we use the maximum degree $K = 10^5$. The dashed and solid lines represent functions $R_1(y)$ and $R_2(y)$, respectively. The triangles and circles mark the critical points and the non-trivial solutions, respectively. (a) $m = 1$ and $\gamma = 2.5$. (b) $m = 1$ and $\gamma = 2.1$. (c) $m = 2$ and $\gamma = 2.5$. (d) $m = 8$ and $\gamma = 2.5$.

that corresponds to the found S_c is $\psi_c^\infty = (S_c)^2 > 0$. The giant cluster size thus undergoes a discontinuous phase transition at z_c^∞. Above the critical point, the fixed point S further grows with z as shown in Fig. 3, corresponding to the percolating state.

2. *Two-layer SF networks*

Now, we study the solution of Eqs. (16) and (17) for infinite generation, for which $x = \mathfrak{F}(px)$ and $\psi = x^2$. For qualitative analysis, supposing p is fixed, and letting $y \equiv pRx$ in Eqs. (16) and

(17), we have

$$R = 1 - G_1(1-y) \equiv R_1(y), \tag{27}$$

$$R = \frac{1}{p}\frac{y}{1-G_0(1-y)} \equiv R_2(y). \tag{28}$$

The solution of these two equations can be represented by the cross points of functions $R_1(y)$ and $R_2(y)$ as shown in Fig. 4. When $y \to 0$, functions $R_1(y)$ and $R_2(y)$ behave as

$$R_1(0) = 0, \quad \left.\frac{dR_1(y)}{dy}\right|_{y=0} = G_1'(1) \to \infty, \tag{29}$$

$$R_2(0) = 0, \quad \left.\frac{dR_2(y)}{dy}\right|_{y=0} \sim \frac{1}{p}\frac{1}{1-G_0(1)} \to \infty. \tag{30}$$

It is not hard to find that $dR_2(y)/dy$ is a higher order infinity, thus $R_2(y)$ is larger than $R_1(y)$ in the area around $y \to 0^+$. In addition, for $y \to 1^-$, $R_1(y)$ and $R_2(y)$ behave as

$$R_1(1) = 1 - \frac{p_1}{z}, \quad \left.\frac{dR_1(y)}{dy}\right|_{y=1} = G_1'(0) = \frac{2p_2}{z}, \tag{31}$$

$$R_2(1) = \frac{1}{p}, \quad \left.\frac{dR_2(y)}{dy}\right|_{y=1} = \frac{1-G_0'(0)}{p} = \frac{1-p_1}{p}. \tag{32}$$

Here $p_1 = c \approx (\gamma-1)m^{\gamma-1}$ (for $m \le 1$) is the fraction of the nodes with degree 1, $p_2 = c2^{-\gamma} \approx (\gamma-1)m^{\gamma-1}2^{-\gamma}$ (for $m \le 2$) is that for degree 2, and $z \approx m(\gamma-1)/(\gamma-2)$ is the average degree.

When $m = 1$, $p_1/z \approx \gamma - 2$, even if $p = 1$, $R_2(y)$ is larger than $R_1(y)$ when $y \to 1^-$. For large γ, such as $\gamma = 2.5$ used in Fig. 4(a), there is no crossing point, meaning that no percolation transition exists for $m = 1$. For comparison, Fig.3(b) shows non-trivial cross points for $\gamma = 2.1$.

When $m > 1$ (*i.e.*, $p_1 = 0$), as shown in Fig. 4(c)-(d), the non-trivial cross points of functions $R_1(y)$ and $R_2(y)$ shift to the right as p grows. The point of tangent with non-zero $R(y)$ and y indicates that the percolation transition is discontinuous. In addition, as m increases, the divergence of $dR_1(y)/dy$ becomes faster when $y \to 0^+$. The tangent point thus tends to the point $(0, 0)$, meaning that a continuous percolation transition can also be found for infinite generation when $m \to \infty$ (average degree is then, naturally, also very large).

II. ALGORITHM

For simulations, our model can be implemented directly as the rules of the model, *i.e.*, performing the percolation process iteratively, whose time complexity is $nO(N)$ for generation n and network size N. To be more efficient, the simulations in this paper are realized by a Leath-like

method [5]. Instead of searching the whole network repeatedly, we evolve nodes (actually with some of its neighbors) one by one to the generation we want to study.

In our algorithm, each node has two variables, generation n_i and root r_i, labeling which generation and cluster node i belongs to. Here, n_i must be non-negative, meaning that node i, as well as some of its neighbors, are in a cluster of generation n_i. In general, $r_i \geq 0$ is the root node of node i (nodes are numbered from 0 to $N-1$). If $-N \leq r_i < 0$, it means that node i is the root of the corresponding cluster and $-r_i$ is the number of nodes in this cluster. Based on this, the root node $root(i)$ of a cluster can be found from any node i in this cluster. One possible way to do this (pseudo code) is as follows [6]:

```
root (i) :
1   j = i
2   while  r_i ≥ 0
3       do
4           r_j = r_i
5           j = i
6           i = r_i
7       end
8   return  i
```

Here, lines 4 and 5 are used to compress the path from node j to the cluster root, which do nothing to this function $root(i)$ itself, but facilitate further searching.

Assuming that we want to obtain the percolation configuration of generation n, the main steps of our algorithm are as follows:

1. Assigning each node a generation $n_i = 0$ and a root $r_i = -N-1$, which means they have not yet been searched.

2. For node i with $n_i < n$, do the following steps:

   a: Determining the network layer l used in generation $n_i + 1$ according to the model setting.

   b: Finding all the nodes (labeled j for convenience) connecting to node i by layer l with $n_j = n_i$ and $root(j) = root(i)$.

c: If the number of the found nodes $N_f = -r_{root(i)}$, let $n_j = n$ for all these nodes including node i; Otherwise, let $r_{root(i)} = -N_f$, and $n_j = n_j + 1$ for all these nodes including node i.

d: If the new $n_i < n$, do steps (a)-(c) again.

3. Do step 2 for all the nodes.

Obviously, this algorithm is more effective than the one that just implementing the model rules directly, since the search times for most nodes are less than n in generation n. Specifically, one can simply use the depth-first or breadth-first searching to implement step 2 of this algorithm. To pursue a more effective way, other typical algorithms in percolation model can also be used to realize step 2, which could depend on the measurement we are interested in. In addition, this type of algorithm has already been used in the percolation on the so-called interdependent networks [7].

III. THE LARGEST CLUSTER AT THE CRITICAL POINTS

As the generation increases, the history-dependent percolation becomes sharper and sharper. This increases the finite-size effect around the critical point, which makes the simulation results appear to deviate from the finite-size scaling of a continuous phase transition. For a better understanding of this finite-size effect, we show the distribution of the order parameter ψ_c^n in individual model realizations in Fig. 5.

For the first several generations (*i.e.*, n is not too large, see for examples Fig. 5(a)–(d)), the values of ψ_c^n congregate in a small region. In these cases, we may not be able to distinguish the non-percolating and percolating realizations, since they all give a ψ_c^n close to zero. In spite of this, the corresponding finite-size scaling still fit well with the theoretical analysis even for small network sizes (see Fig.2(b) in main text). However, with the increasing of n, the percolation transition becomes sharper and sharper, which produces excessively high percolation rates at the critical point for small systems. The results are the distribution of ψ_c^n becomes broader and strongly bimodal for late generations ($n \gtrsim 20$), and the value obtained by averaging over these ψ_c^n become larger than expectations. Therefore, the deviation from the finite-size scaling for large n is caused by the consideration of these excessively percolating realizations.

When $n = \infty$ where the discontinuous transition exists, although theoretically the order parameter ψ_c^∞ should be non-zero at the critical point, a finite system at (even above) the critical point

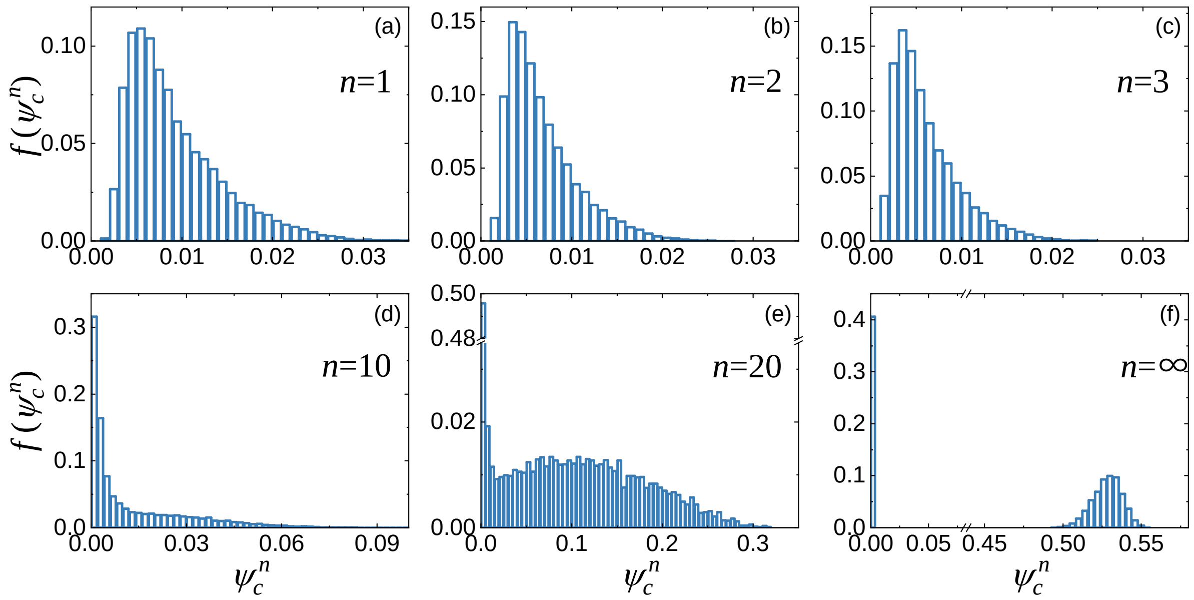

FIG. 5. The distribution of the order parameter ψ_c^∞ at the critical point. The size of the network used in the simulation is $N = 2^{20}$.

can also fail to percolate and thus the bimodal distribution of ψ_c^∞ is still present. The prominent bimodal distribution of ψ_c^∞ (see Fig. 5(f)) allows the non-percolating and percolating realizations to be distinguished: ψ_c^∞ close to zero for the non-percolating realizations and larger values for percolating realizations. If the evaluation of simulations is limited to the percolating realizations, the expected behavior of ψ_c^∞ is again recovered (see Fig.2(d) in main text).

IV. SOCIAL NETWORK

In Fig. 6, we apply our model on a social network composed of users who are active on both Twitter and FriendFeed. Among the 150,684 common users of the two networks, there are 8,308,326 and 5,270,665 links in the Twitter and FriendFeed layer, respectively.

V. BRAIN NETWORK

In Fig. 7, we give the pattern of the remaining nodes and links (the giant cluster of infinite generation) of HC and MDD participants when p is slightly larger than p_c. We can find that for $z = 6$ the remaining nodes and links of MDD participant is more sparse and dispersed, and mainly located in frontal, parietal, and occipital lobes, and no obvious community structure formed.

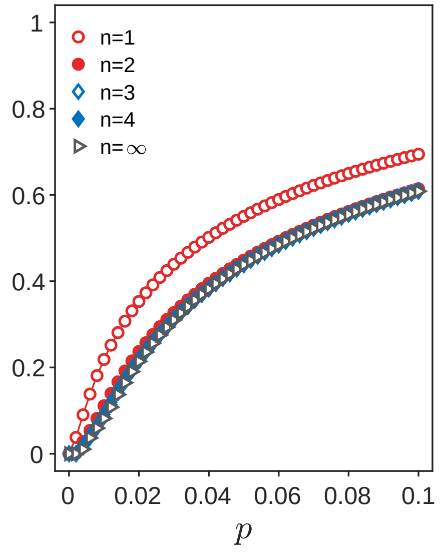

FIG. 6. History-dependent percolation on social network composed of users who are active on both Twitter and FriendFeed. The average degrees are $z \approx 110$ and 70 in the two network layers, respectively. Here p is the link occupation probability.

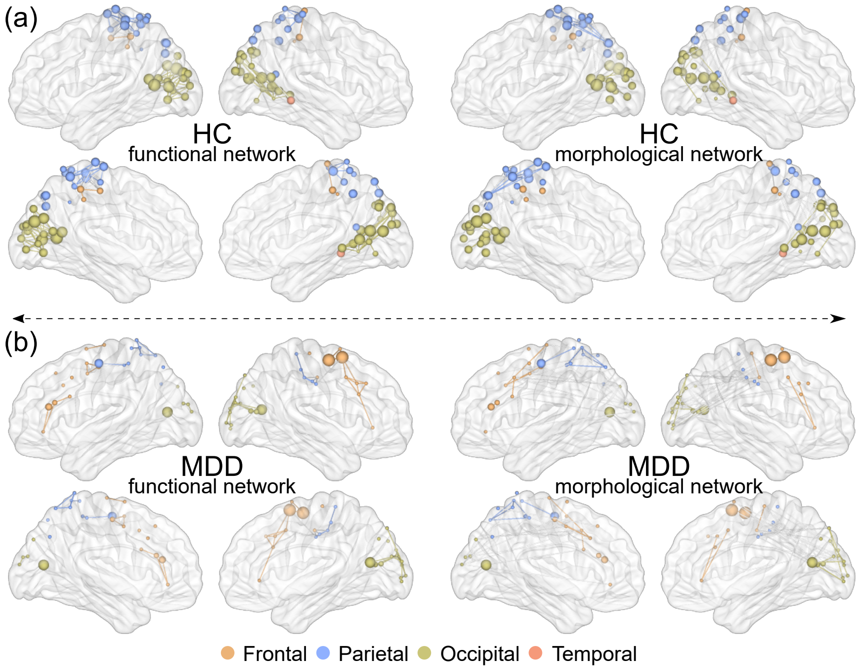

FIG. 7. The visualization of human brain two layers networks at degree $z = 6$ and 1024 parcellation templates when p is slightly larger than the corresponding p_c. (a) The HC participant's bilayer brain network. (b) The MDD participant's bilayer brain network.

VI. MRI DATA AND PROCESSING

We selected a healthy control (HC) participant (subject ID:206525) and obtain the corresponding resting-state functional magnetic resonance imaging (R-fMRI) data and T1-weighted data from the “S1200” release of the Human Connectome Project data (1U54MH091657) [8]. We similarly obtained the R-fMRI and T1-weighted data of one major depressive disorder (MDD) participant from our own dataset for comparison. The entire processing of the R-fMRI data is conducted using the Statistical Parametric Mapping (SPM12, version r7219) [9] and our own MATLAB codes. We processed the T1-weighted data with the Computational Anatomy Toolbox (CAT12, version r1278) [10].

R-fMRI data processing. In the current study, our R-fMRI data processing includes the following steps: 1) Removing the volumes of the first 10 seconds; 2) Realignment of all volumes to the first volume; 3) Mean-based intensity normalization; 4) Spatial normalization to the MNI template with EPI ($2 \times 2 \times 2mm$ voxel size); 5) Linear detrending of retain mean and bandpass filtering between 0.043 and $0.087Hz$ using the Butterworth filter; 6) Denoising: 24HMP+8Phys+4GSR+Spikereg [11]; 7) Each fMRI voxel value is weighted by the gray matter probability.

T1-weight data processing. The raw MRI data were checked manually to ensure no obvious artifacts. We use the CAT12 toolbox to perform the voxel-based morphometry analysis and the T1-weighted image is segmented into gray matter (GM), white matter and cerebrospinal fluid. The resulting GM images are normalized to the MNI space and undergo nonlinear modulation. Finally, the GM volume images for each participant are obtained.

---

[1] William Aiello, Fan Chung, and Linyuan Lu. A random graph model for power law graphs. *Experimental Mathematics*, 10(1):53–66, 2001.

[2] Reuven Cohen, Daniel ben Avraham, and Shlomo Havlin. Percolation critical exponents in scale-free networks. *Phys. Rev. E*, 66:036113, 2002.

[3] D.-S. Lee, K.-I. Goh, B. Kahng, and D. Kim. Evolution of scale-free random graphs: Potts model formulation. *Nucl. Phys. B*, 696(3):351 – 380, 2004.

[4] J. Gao, S. V. Buldyrev, S. Havlin, and H. E. Stanley. Robustness of a network of networks. *Phys. Rev. Lett.*, 107:195701, 2011.

[5] P. L. Leath. Cluster size and boundary distribution near percolation threshold. *Phys. Rev. B*, 14:5046–5055, 1976.

[6] M. E. J. Newman and R. M. Ziff. Fast monte carlo algorithm for site or bond percolation. *Phys. Rev. E*, 64:016706, 2001.

[7] P. Grassberger. Percolation transitions in the survival of interdependent agents on multiplex networks, catastrophic cascades, and solid-on-solid surface growth. *Phys. Rev. E*, 91:062806, 2015.

[8] D. C. Van Essen, S. M. Smith, D. M. Barch, T. E. J. Behrens, E. Yacoub, and K. Ugurbil. The WU-minn human connectome project: An overview. *NeuroImage*, 80:62–79, 2013.

[9] http://www.fil.ion.ucl.ac.uk/spm/software/spm12.

[10] http://www.neuro.uni-jena.de/cat12.

[11] L. Parkes, B. Fulcher, M. Yücel, and A. Fornito. An evaluation of the efficacy, reliability, and sensitivity of motion correction strategies for resting-state functional mri. *NeuroImage*, 171:415–436, 2018.
